# Natural products from filamentous fungi and production by heterologous expression

**DOI:** 10.1007/s00253-016-8034-2

**Published:** 2016-12-13

**Authors:** Fabrizio Alberti, Gary D. Foster, Andy M. Bailey

**Affiliations:** 1 School of Life Sciences and Department of Chemistry, University of Warwick, Gibbet Hill Road, Coventry, CV4 7AL UK; 2 School of Biological Sciences, University of Bristol, 24 Tyndall Avenue, Bristol, BS8 1TQ UK

**Keywords:** Fungi, Heterologous expression, Natural products, Gene clusters, Secondary metabolites

## Abstract

Filamentous fungi represent an incredibly rich and rather overlooked reservoir of natural products, which often show potent bioactivity and find applications in different fields. Increasing the naturally low yields of bioactive metabolites within their host producers can be problematic, and yield improvement is further hampered by such fungi often being genetic intractable or having demanding culturing conditions. Additionally, total synthesis does not always represent a cost-effective approach for producing bioactive fungal-inspired metabolites, especially when pursuing assembly of compounds with complex chemistry. This review aims at providing insights into heterologous production of secondary metabolites from filamentous fungi, which has been established as a potent system for the biosynthesis of bioactive compounds. Numerous advantages are associated with this technique, such as the availability of tools that allow enhanced production yields and directing biosynthesis towards analogues of the naturally occurring metabolite. Furthermore, a choice of hosts is available for heterologous expression, going from model unicellular organisms to well-characterised filamentous fungi, which has also been shown to allow the study of biosynthesis of complex secondary metabolites. Looking to the future, fungi are likely to continue to play a substantial role as sources of new pharmaceuticals and agrochemicals—either as producers of novel natural products or indeed as platforms to generate new compounds through synthetic biology.

## Introduction

### Bioactive natural products from filamentous fungi

**Fig. 1:**
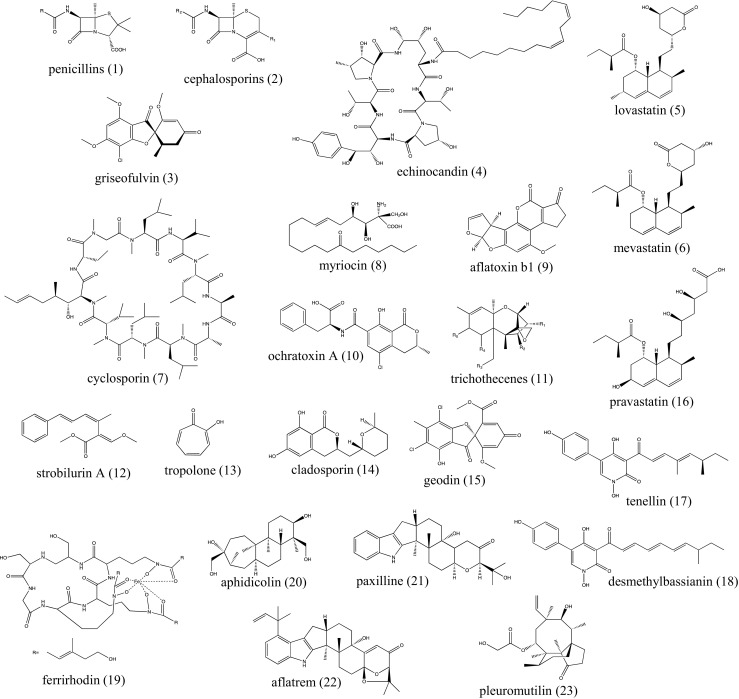
Chemical structures of all fungal bioactive natural products mentioned in this article

Fungi and other microorganisms represent an invaluable source of natural product (NP) bioactive compounds (Fig. 1), which are exploited in various contexts, ranging from crop protection to human medicine. In the last decades, drug discovery has been greatly prompted by the increasing number of whole-genome sequences that have become available, which exposed a myriad of putative gene clusters for potentially bioactive compounds. The approach quickly expanded from studies on bacteria to fungi as sequencing costs dropped and the technology improved. Among fungal natural products, particular interest is given to antimicrobials, due to the reduction in effectiveness of existing antibiotics used to treat bacterial infections, which is seen as a major threat to global health security (Aiken et al. 2014). Many prominent examples of compounds that affect our everyday life fall into the group of fungal secondary metabolites (SMs) (Hoffmeister and Keller 2007). Penicillins (1) and cephalosporins (2), for instance, are *β*-lactam antibiotics and represent the most widely used antimicrobials in the world: cephalosporins accounting for 28% ($11.9 billion in 2009) and penicillins for 19% ($7.9 billion in 2009) of the global market of antibiotics (Hamad 2010). In addition to antibacterial compounds, there are a number of fungal-derived antifungals such as griseofulvin (3) and echinocandin (4) which have been exploited for use in medicine. Another medical application that fungal SMs are known for is that of cholesterol-lowering agents, such as the statins, some of which act through selective inhibition of squalene synthase and some others by hindering the activity of HMG-CoA reductase, the first enzyme involved in biosynthesis of cholesterol (Istvan and Deisenhofer 2001). This class of compounds includes lovastatin (5), which is primarily produced by *Aspergillus terreus*, and mevastatin (6), found in *Penicillium citrinum* (Manzoni and Rollini 2002). Some other SMs produced from fungi can have immunosuppressant activity. An example is given by the non-ribosomal peptide (NRP) cyclosporin (7), produced by *Tolypocladium niveum* and widely used to avoid organ rejection in transplant surgery (Weber et al. 1994). Other fungal-derived compounds even display activities against multiple sclerosis, such as fingolimod, a chemical derivative of the NP myriocin (8) (Cohen et al. 2010).

Mycotoxins are also produced by fungi, as an example of NPs whose presence as contaminants in food processing is highly feared and monitored. Among the best known mycotoxins, we find the aflatoxins (9), produced by *Aspergillus flavus* (Hesseltine et al. 1966), the ochratoxins (10) from *Aspergillus* and *Penicillium* spp. (Kuiper-Goodman and Scott 1989), and the trichothecenes (11), found in *Fusarium* sp. and other species (Sudakin 2003).

In some cases, fungal SMs may not be used directly as they are found in nature, but be subjected to derivatisation to give semisynthetic derivatives such as the enormous diversity of the various penicillins, all of which start from the fungal fermentation product. Alternatively, such secondary metabolites may serve as a model for the development of completely synthetic derivatives. This is the case of the polyketide-based strobilurin A (12) isolated from the basidiomycete *Strobilurus tenacellus* (Anke et al. 1977), which inspired the discovery and exploitation of *β*-methoxy-acrylic acid, forming the basis of the strobilurin fungicides. This class of antifungals includes one of the world’s most sold fungicides, azoxystrobin (Bartlett et al. 2002).

### Natural product gene clusters

SMs are usually produced by fungi through the catalytic activity of one or more enzymes or sometimes through the activity of large multifunctional polypeptides. When several enzymes cooperate together towards the biosynthesis of a SM, the corresponding genes tend to be forming a gene cluster (Keller and Hohn 1997; Yu and Keller 2005; Osbourn 2010). Such a situation was well known in bacteria and has become regarded as the normal situation in fungi, and there is increasing evidence that the same principle of gene clusters sometimes applies to plant secondary metabolic pathways (Nützmann and Osbourn 2014). Clustering or co-localisation of the genes for a particular product may occur for a number of reasons. Those currently most favoured are that it helps facilitate coordinated regulation of all the genes by having them all in a similar physical location. Another hypothesis is that it helps facilitate transmission of the entire pathway, either through sexual transmission or indeed through horizontal transfer.

Clustering of genes that cooperate towards biosynthesis of a NP greatly aids researchers in characterising and even directing biosynthesis of the corresponding metabolite. Gene clusters can be searched for within assembled genomes using a range of bioinformatics tools, as has been widely reviewed elsewhere (Weld et al. 2006; Brakhage and Schroeckh 2011; Wiemann and Keller 2013; Yaegashi et al. 2014). Once a gene cluster is identified, its full characterisation, as well as production in high yields of the corresponding specialised metabolite, can be achieved through heterologous expression (Lazarus et al. 2014). This consists of cloning one or more genes from a donor species and introducing them in a heterologous (secondary) host. Heterologous expression is considered to be the best available option when pursuing characterisation of biosynthetic genes in fungi that show slow growth in laboratory conditions and have scarce availability of genetic tools (Schmidt-Dannert 2015). Furthermore, it is often used as a tool to increase production yields of bioactive NPs, frequently representing a more profitable alternative to total synthesis. A certain degree of flexibility is encountered when choosing which secondary host to use for heterologous expression of the gene cluster of interest, ranging from unicellular model organisms to well-characterised filamentous fungi.

## Heterologous expression in *Escherichia coli* and yeast hosts

One of the first successes for heterologous expression of fungal secondary metabolite genes was that of 6-methylsalicylic acid synthase (6-MSAS) from *Penicillium patulum* in *Streptomyces coelicolor* (Bedford et al. 1995). Most research however has focused on *E. coli*, given it is the best model prokaryotic host for protein expression and purification. It has also been used to recreate biosynthetic pathways for metabolites of interest or to simply express a few genes from a cluster for partial recreation of a pathway. Usually, the compounds that have been heterologously produced in *E. coli* are of bacterial origin. In this context, a hallmark in heterologous production of complex non-ribosomal peptide-polyketide hybrid (NRP-PK) natural products was established by Pfeifer et al. (2003), who recreated complete biosynthesis of yersiniabactin (Ybt) in *E. coli*, upon heterologous expression of its gene cluster in a previously engineered strain of the model organism, which was able to support correct post-translational processing of the synthase enzyme (Pfeifer et al. 2001). The results with expression of fungal secondary metabolite genes in *E. coli* have been somewhat mixed; whilst some groups have reported success, such as expression of 6-MSAS (Kealey et al. 1998), other research groups have had only limited (if any) success with this system, particularly where expression of very large proteins such as non-ribosomal peptide synthetases (NRPSs) or polyketide synthases (PKSs) are concerned.

*E. coli* has many restrictions that prevent its use to recreate entire fungal SM pathways. Firstly, this host has mainly been used as a model organism and as a producer of heterologous proteins, but it has not been developed as a SM secretion system. *E. coli* cannot usually support post-translational modifications that occur after protein synthesis and are required for functionality of some enzymes. This is particularly important when the core synthases such as PKSs and NRPSs need activation by phosphopantetheinyl transferase or when cytochrome P450 oxidoreductases need suitable redox partners. An added complication is that *E. coli* has a different codon usage than higher fungi, therefore sometimes requiring codon optimisation before expression of the heterologous genes. Moreover, production of antibiotics in *E. coli* may be challenging as the host could be susceptible to the desired NP.

There have however been several examples of terpene cyclases being expressed in *E. coli* where just one fungal enzyme needed to be expressed and without any post-translational modification (Schmidt-Dannert 2015). A more common way of using *E. coli* to synthesise heterologous NPs is through purification of the enzymes upon expression of foreign genes, followed by incubation of the purified protein with its substrate in an in vitro reaction. This can be useful when pursuing functional characterisation of biosynthetic genes in a clean background. For example, Davison et al. (2012) expressed the genes of the tropolone gene cluster from *Penicillium stipitatum* in *E. coli* and purified the correspondent proteins. The product of the gene *tropC*, a non-heme Fe(II)-dependent dioxygenase, was used to catalyse in vitro conversion of 3-methylorcinaldehyde into tropolone (13), therefore revealing the catalytic activity of this enzyme.

The yeast *Saccharomyces cerevisiae* is a unicellular model ascomycete fungus with a long history of exploitation for biotechnology, having been employed in many cases as a recipient for production of heterologous molecules and recreation of biosynthetic pathways for metabolites of interest. The most prominent example so far is perhaps the production of the sesquiterpene artemisinic acid, the precursor of the plant-derived antimalarial drug artemisinin (Ro et al. 2006).

Several examples of heterologous biosynthesis of NPs from higher fungi in yeast are also found in literature. Ishiuchi et al. (2012) for instance developed a yeast-based platform for heterologous expression of PKS and NRPS genes from higher fungi. This encompasses an ad hoc-engineered strain of *S. cerevisiae* and an expression vector that can be assembled with the PKS or NRPS gene of interest using overlap extension PCR and yeast-based homologous recombination, which aid cloning of large synthase and synthetase genes (typically from 5 to 20 kb). In this work, the products of five PKSs and one NRPS from three different fungi were produced heterologously and characterised. Furthermore, a previously uncharacterised NRP was identified, showing the applicability of this system to genome mining (Ishiuchi et al. 2012). More recently, Cochrane et al. (2016) have had success expressing individual and multiple PKSs from the lichen fungus *Cladosporium cladosporioides* to identify the enzymes responsible for synthesis of the antimalarial cladosporin (14).

As with *E. coli*, the yeast *S. cerevisiae* has also been employed for purification of complex enzymes and consequent characterisation of their catalytic activity through in vitro reactions. For instance, Ma et al. (2009) purified the highly reducing iterative polyketide synthase LovB (lovastatin nonaketide synthase) from *A. terreus*, upon heterologous expression in *S. cerevisiae*, and characterised the catalytic activity of this megasynthase, as well as of its partner enoyl reductase enzyme LovB.

Enzymes of fungal origin are also produced using yeast as heterologous host, as shown by Vaquero et al. (2015), who produced a versatile sterol-esterase, active for both synthesis and hydrolysis of triglycerides, from the fungus *Ophiostoma piceae*. Ultimately, advantages of using the yeast *S. cerevisiae* as a heterologous expression system are various: it is a unicellular organism and has rapid growth, there are plenty of tools available for genetic engineering, as a eukaryote, it can typically support protein folding, and most, but not all, post-translational modifications were required for functionality of eukaryotic proteins. The yeast *S. cerevisiae* has also been given the status of generally regarded as safe (GRAS) organism, as it is not producing any known toxic or oncogenic product.

## Heterologous expression in filamentous fungi

Besides *E. coli* and *S. cerevisiae*, filamentous fungi are often chosen when complete biosynthesis of a fungal SM is pursued. They usually have very simple growth requirements and are often amenable to large-scale fermentation; indeed, there is considerable experience in optimising the cultivation of fungi such as *Penicillium chrysogenum*. Among the various fungi, *Aspergillus* species are the most commonly used secondary hosts. *Aspergillus nidulans* is a genetic model species among filamentous fungi and has been used also as heterologous host to study gene clusters from other species. Nielsen et al. (2013) developed a smart system for selectable marker recycling in this host based on homologous integration in the *IS1* locus, which supports high levels of expression. This approach allowed them for a stepwise transfer of all the 13 genes of the geodin (15) cluster from *A. terreus* into *A. nidulans*, enabling to dissect the biosynthesis of the NP in the secondary host. A more complex synthetic biology approach for production of the cholesterol-lowering agent pravastatin (16) was reported by McLean et al. (2015). The authors used *P. chrysogenum* to express the original compactin gene cluster from *P. citrinum*, along with the gene *CYP105AS1*—encoding a cytochrome P450—from *Amycolatopsis orientalis* (also known as *Streptomyces orientalis*), converting the compactin to pravastatin. The *P. chrysogenum* host they used was a strain that had been derived from a commercial penicillin-producing strain and had subsequently been manipulated to lose its penicillin gene cluster. This host had therefore been through multiple rounds of conventional strain improvement, optimising its secondary metabolism potential under fermentation conditions. This is also an example of how heterologous platforms can be used to direct the biosynthesis towards a derivative of the NP, bypassing or reducing the need for expensive synthetic derivatisation. Besides recreating total biosynthesis of SMs, heterologous expression has also the potential of reprogramming the activity of PKSs and other synthase enzymes, as shown by Fisch et al. (2011). In this work, rational domain swaps were performed between the PKSs for tenellin (17) and desmethylbassianin (18), followed by heterologous expression of the hybrid synthases in *A* *spergillus oryzae*, and consequent isolation of compounds where differences in chain length and methylation pattern could be linked to the corresponding domain swaps.

*A. oryzae* is a species that has been used in many cases for heterologous expression. This species is of particular interest as it has a long history in food technology, since it has been used for several centuries for the fermentation of cereals and legumes to get commonly used food products like sake, miso, and soy sauce, and it has been given the status of GRAS organism, therefore safe to be used for the production of enzymes and metabolites intended for human use (Barbesgaard et al. 1992). *A. oryzae* also benefits from being taxonomically close to the genetic model *A. nidulans*, and many of the transformation markers and regulatory elements in expression vectors can be used in both species. Molecular tools have been established to manipulate *A. oryzae*, such as selectable markers and promoters that can ensure high levels of constitutive or inducible expression of foreign genes (Yamada et al. 1997; Jin et al. 2004a, b; Pahirulzaman et al. 2012), which in turn can allow for high yields of heterologous production of bioactive NPs (Table 1). For instance, the biosynthesis of the polyketide tenellin (17) from the entomopathogenic ascomycete *B* *eauveria bassiana* was reproduced in *A. oryzae* (Heneghan et al. 2010). Four genes, including a gene for an iterative PKS fused to a single module NRPS, were inserted into *A. oryzae* by employing three vectors. Each gene was flanked by the promoter *PamyB* and the terminator *TamyB* from the *Taka-amylase A* gene from *A. oryzae*, whose enzymatic product degrades starch into dextrin (Tada et al. 1989); expression of this gene is induced by starch; hence, its promoter is often used to drive inducible expression in *A. oryzae*. The authors reported a titre of tenellin of 243 mg L^−1^ in *A. oryzae* (Heneghan et al. 2010), which was more than fivefold higher than the production reported for the native host. The use of promoters with high level of expression derived from the secondary host is likely to have played an important role in the high yield of heterologous product obtained. Munawar et al. (2013) used *A. oryzae* to heterologously express the NRPS responsible for synthesis of the siderophore ferrirhodin (19) from *Fusarium sacchari*. In other works, Fujii et al. (2011) expressed four genes from a gene cluster of the plant pathogenic fungus *Phoma betae* in *A. oryzae*, achieving production of the antibiotic diterpene aphidicolin (20). A step-by-step transformation of *A. oryzae* with the four genes of the aphidicolin cluster allowed the authors to isolate the intermediates produced along the pathway and characterise the catalytic activity of the enzymes involved*.* In this case, the complementary DNA (cDNA) of the four putative biosynthetic genes was used, therefore avoiding potential problems related with the maturation of mRNA in *A. oryzae*. The approach is not always straight forward; *A. oryzae* may encounter problems with intron splicing, as seen during heterologous expression of the *ACE1* gene from *Magnaporthe oryzae* (Song et al. 2015), as well as for 3-methylorcinaldehyde synthase of *Acremonium strictum* (Bailey et al. 2007). In other works, Tagami et al. (2013) successfully synthesised the indole-diterpene paxilline (21) in *A. oryzae*, a bioactive natural product that shows MRSA growth inhibition. Six genes had first been identified as being involved in biosynthesis of paxilline in the native producer *Penicillium paxilli* (Saikia et al. 2006, 2007) and were expressed heterologously by Tagami et al. (2013). Also in this case, the authors adopted a stepwise transformation approach and were therefore able to isolate and identify the intermediate compounds of the paxilline biosynthetic pathway. Ultimately, a yield of 35 mg L^−1^ of the final product was reported in *A. oryzae*. Adopting a similar approach, the same research group also recreated biosynthesis of aflatrems (22), indole-diterpene compounds with similar structure and properties to those of paxilline (Tagami et al. 2014). Insertion of the first four genes of the pathway from the original host *A. flavus* into *A. oryzae* allowed them to achieve production of paspaline, a common precursor to aflatrems, paxilline, and other indole-diterpenes. Further introduction of the remaining three genes of the gene cluster led to biosynthesis of the two final products aflatrem and β-aflatrem.

**Table 1:** Reported yields of heterologous production of bioactive fungal natural products within the secondary host *Aspergillus oryzae*

Natural product	Native host	Bioactivity	Reported yield^a^	References
Citrinin	*Monascus purpureus*	Antibacterial	1.48 mg L^−1^	Sakai et al. (2008)
Tenellin	*Beauveria bassiana*	Inhibitor of erythrocyte membrane ATPase activity; iron chelator	243 mg L^−1^	Heneghan et al. (2010)
Aphidicolin	*Phoma betae*	Inhibitor of DNA polymerase α	0.33 mg L^−1^	Fujii et al. (2011)
Paxilline	*Penicillium paxilli*	Inhibitor of the high-conductance calcium-activated potassium channel; antibacterial	35 mg L^−1^	Tagami et al. (2013)
Aflatrem	*Aspergillus flavus*	Tremorgenic	54 mg Kg^−1b^	Tagami et al. (2014)
Pleuromutilin	*Clitopilus passeckerianus*	Antibacterial	84 mg L^−1^	Bailey et al. (2016)

^a^The yield in the heterologous host is expressed as mg of compound produced per litre of liquid medium

^b^The yield, in this case, is expressed as mg of compound per Kg of rice, as the heterologous host was grown in a solid medium made of polished rice (100 g) and adenine (10 mg) (Tagami et al. 2014)

Another example of heterologous production of a fungal SM in *A. oryzae* was recently reported by Bailey et al. (2016), who recreated total biosynthesis of the antibiotic pleuromutilin (23), a diterpene compound naturally produced by *Clitopilus passeckerianus* and related species (Hartley et al. 2009). Despite the first developing a protocol for transformation of the producing fungus (Kilaru et al. 2009), heterologous expression of the putative gene cluster in the secondary host *A. oryzae* was preferred over manipulation within the native producer, which is a known dikaryotic species. The expression of the cDNA of the putative biosynthetic genes allowed accumulation of pleuromutilin, as well as another known product of fermentation of *C. passeckerianus*, 14-O-acetyl-mutilin (Bailey et al. 2016). The expression vectors developed by Pahirulzaman et al. (2012) were employed in this case, where strong constitutive promoters derived from genes involved in the primary metabolism drive expression of the genes of interest. A remarkable tenfold increase in antibiotic production compared to the native host producer (final yield in *A. oryzae* 84 mg L^−1^) could be reported. Following up on this work, the biosynthetic pathway for the antibiotic was also dissected in the same secondary host, allowing to describe the catalytic activity of the enzymes involved (Alberti et al. 2016). Worth mentioning is that for the first time, heterologous total biosynthesis of a SM from a basidiomycete fungus was recreated in an ascomycete secondary host, opening the way for characterisation of other basidiomycete NPs in better-known and more amenable hosts. As with many other complex fungal SMs, total chemical synthesis of pleuromutilin has only been achieved with extremely low yields (0.7% overall yield to the final product in a 34-step synthesis), as reported by Fazakerley et al. (2013). Due to the energy consumption and high costs of the catalysts used, large-scale production of pleuromutilin and other complex fungal NPs through total synthesis is not economically feasible. Indeed, total biosynthesis of these bioactive compounds through heterologous expression represents a far more appealing and inexpensive alternative.

## Conclusions

Filamentous fungi can produce a wide range of SMs, which have been exploited as pharmaceuticals for decades (Hoffmeister and Keller 2007). Heterologous expression has been established as a potent approach both for characterising cryptic gene clusters from filamentous fungi and for achieving production of bioactive NPs in a clean background, facilitating their purification and downstream applications.

In this review, the most common platforms for heterologous biosynthesis of fungal NPs were analysed. Unicellular hosts, *E. coli* in particular, have only rarely been used to recreate whole biosynthetic pathways from fungi, despite representing a suitable platform for purification and study of individual enzymes. In contrast, plenty of tools have been developed for the genetic manipulation of *Aspergillus* and other filamentous fungi used as heterologous hosts, going from recycling of selectable markers (Nielsen et al. 2013) to multigene expression vectors (Pahirulzaman et al. 2012). When the genome of the species of interest has been sequenced and assembled, synthetic gene sequences, based on the most likely predicted coding sequence for the genes under study, can nowadays be obtained rather inexpensively. These can consequently be assembled in suitable vectors for constitutive or inducible expression in the preferred filamentous fungus heterologous host, making it unnecessary to even culture the donor organism. Given the successful examples of biosynthetic pathways recreated in the last years through heterologous expression, it can be envisaged that this will enable characterisation of many other SMs from fungi in the future not only for the production of naturally occurring compounds but also for refactoring of metabolic pathways and consequent production of novel metabolites through synthetic biology.
